# Effect of high carbohydrate diet on elongase and desaturase activity and accompanying gene expression in rat’s liver

**DOI:** 10.1186/s12263-017-0551-9

**Published:** 2017-01-25

**Authors:** Jagoda Drąg, Anna Goździalska, Małgorzata Knapik-Czajka, Anna Gawędzka, Katarzyna Gawlik, Jerzy Jaśkiewicz

**Affiliations:** 1 Department of Analytical Biochemistry, Faculty of Pharmacy, Jagiellonian University Medical College, Krakow, Poland; 2 Faculty of Health and Medical Sciences, Andrzej Frycz Modrzewski Krakow University, Krakow, Poland; 3 Department of Diagnostics, Chair of Clinical Biochemistry, Jagiellonian University Medical College, Krakow, Poland; 4 Andrzej Frycz Modrzewski Krakow University, 1 G. Herlinga-Grudzińskiego St., 30-705 Krakow, Poland

**Keywords:** Elongases, Desaturases, Fatty acids, High carbohydrate diet

## Abstract

**Background:**

Hepatic fatty acids (FAs) are modified through different metabolic pathways including elongation and desaturation. These processes are catalyzed by elongases and desaturases, respectively. Glucose, by transcription factors, regulates these processes. The aim of the study was to evaluate the influence of high carbohydrate diet (68%) on the expression of elongase (Elovl-2, Elovl-5, and Elovl-6) and desaturase (∆5D, ∆6D, Scd 1, Scd 2) genes and the activity of the enzymes. The changes in serum lipid profile (triglycerides (TG), total cholesterol (TC), HDL cholesterol) and glucose concentration were measured. Male Wistar rats were randomized into two study groups: animals fed with high carbohydrate diet (*n* = 6; HiCHO) and a control group fed with a standard diet (*n* = 6; ST). The expression of mRNA was determinate using reverse transcription PCR (RT-PCR). Hepatic FA composition was determined by gas chromatography, and FA ratios were used to estimate the activity of enzymes. Serum lipid profile and glucose concentration were measured using spectrophotometric methods.

**Results:**

The mean values of transcript expression of all examined elongases and desaturases in liver HiCHO rats were higher as compared to ST. Higher expression did not always correspond to higher activity (as index). More monounsaturated FAs (MUFAs) were detected in the liver of HiCHO rats as compared to ST. Serum TG level was higher in the HiCHO than in ST.

**Conclusions:**

These studies support the notion that the regulation of both Elovl and desaturase expression may play an important role in managing hepatic lipid composition in response to changes in dietary status.

## Background

In mammals, the liver is responsible for the conversion of excess dietary carbohydrates into triglycerides (TG), through de novo lipogenesis (DNL). Fatty acids (FAs) derived from a diet as well as synthesized from carbohydrates are modified through different metabolic pathways including elongation and desaturation [1]. FAs are then used mainly for TG synthesis, then incorporated into lipoproteins (mainly VLDL), and released to plasma [2–4]. Parts of FAs are the components of phospholipids (PL) and neutral lipids [5].

FA elongation is performed in four steps, catalyzed by the following enzymes: ketoacyl-CoA synthase (elongase, EC 2.3.1.), 3-ketoacyl-CoA reductase (KAR; EC 1.1.1.-), 3-hydroxyacyl-CoA dehydratase (EC 4.2.1.134), and 2,3-transenoyl-CoA reductase (TER; EC 1.3.1.38). Elongation occurs in the membrane of the endoplasmic reticulum (ER) with malonyl-CoA as two-carbon donor, mitochondria with acetyl-CoA as two-carbon donor and peroxisomes [2, 6–8]. Based on different substrate specificity, elongases (Elovl) can be divided into four groups. The first group are isoforms that elongates saturated (SFA) and monounsaturated fatty acids (MUFA) (Elovl-1, Elovl-3, Elovl-6, and Elovl-7); the second group includes elongase 2 (Elovl-2) that is involved in polyunsaturated fatty acid (PUFA) synthesis; the third group is elongase 5 (Elovl-5) catalyzing elongation of 18–22 carbons of PUFA as well as 16:1 n-7, and the last one is elongase 4 (Elovl-4) that elongates saturated as well as unsaturated very long chain FAs [4, 7–10].

FAs are also products in the oxidative desaturation process. The introduction of a double bond into a carbon chain occurs in the endoplasmic reticulum and requires molecular oxygen, NAD(P)H, flavoprotein cytochrome b5 reductase, and the electron acceptor cytochrome b5 and is catalyzed by a specific desaturase. In mammals, FA desaturases (EC 1.14.19) are acyl-CoA desaturases, which include mainly ∆5 desaturase (∆5D), ∆6 desaturase (∆6D), and ∆9 desaturase (∆9D) (also known as stearoyl-CoA-desaturase or SCD). ∆5D and ∆6D catalyze the biosynthesis of PUFA. Their most common substrates are C16–C24, mainly exogenous nutrients as α-linoleic acid and linolenic acid. ∆9D catalyzes introduction of a double bond into SFA with the preferred substrates as C16:0-CoA and 18-CoA. Two isoforms of ∆9D called Scd1 and Scd2 exist in rats [2, 5, 11, 12].

Physiological control of FA elongation and desaturation remains poorly defined. It is known that the elongases and desaturases appear to be mainly regulated on the transcriptional level. It was proposed that transcriptional factors such as carbohydrate-responsive element-binding protein (ChREBP) [3, 13–17], liver X receptor α (LXRα) [2, 18, 19], and sterol regulatory element-binding proteins 1c (SREBP-1c) [3, 8, 20–23] are key mediators in elongases and desaturases messenger RNA (mRNA) levels regulation. These transcription factors are modified by different nutritional factors and metabolic states. It has been shown that glucose as a substrate for FA synthesis induces lipogenesis and expressions of lipogenic genes [24]. In primary hepatocytes, Elovl-6 and SCD mRNAs were induced by glucose [3]. Expression of the murine liver stearoyl-CoA desaturase gene (SCD1) is induced upon feeding fasted mice a fat-free, high carbohydrate diet [25]. Expression and activity of elongases and desaturases was changed in adult male rats by HiCHO diet supplemented with olive oil (10%, *w*/*w*), fish oil (10%, *w/w*), or the PPAR-agonist WY14, 643 (0.1%, *w*/*w*) [1]. In the liver of diabetic rats (diabetes induced with streptozotocin), declined Elovl-6 and SCD mRNA abundance were shown [1]. Treatment of rat insulinoma (INS)-1 cells with elevated glucose increased de novo FA synthesis by modification of elongases and desaturases expression and enhanced production of monounsaturated fatty acids [4].There are no studies showing the changes of mRNA levels for many elongases and desaturases together with their activities (expressed as indexes) in Wistar’s rat livers after the carbohydrate diet (68% sucrose).

The aim of the present study was to evaluate the influence of high carbohydrate diet (68%) on the expression of elongase (Elovl-2, Elovl-5, and Elovl-6) and desaturases’ (∆5D, ∆6D, Scd 1, and Scd 2) genes. The activities of the ∆5D (20:4n-6/20:3n-6), ∆6D (18:3n-6/18:2n-6), stearoyl-CoA desaturase 1 (SCD-1; 16:1n-7/16:0, SCD-16 and 18:1n-9/18:0, SCD-18), DNL (16:0/18:2n-6), Elovl-6 (18:0/16:0) and FA elongation (18:0 + 18:1 n-9/16:0) was expressed as FA ratios. For the first time, the ratio 20:3 n-6/18:3 n-6 was used as the activity of Elovl-5 (Elovl-5 index). FA composition in rat livers was determined. We have also measured the changes in serum lipid profile (TG, total cholesterol (TC), HDL cholesterol) and glucose concentration.

## Methods

### Animals

Animal experiments were conducted in accordance with the guidelines for animal experiments of Animal Research Committee and were approved by the Jagiellonian University Ethic Committee.

Male Wistar rats weighing 151 ± 3 g were purchased from the breeding facility of the Jagiellonian University Faculty of Pharmacy. Animals were multi-caged and maintained under standardized conditions of artificial 12-h light/dark cycle and constant room temperature (21–23 °C). Animals were given ad libitum access to food and water. Animals were randomized into two study groups: (1) standard diet-fed controls (ST) (*n* = 6, control) (Murigran, Concentrate and Mix Feed Factory AGROPOL, Motycz) and (2) high-carbohydrate diet-fed study group (HiCHO) (*n* = 6) (containing 68% carbohydrates, MP Biomedicals, 960236), until rats achieved 250 g (25 days ST vs. 21 days HiCHO). Then rats were sacrificed, and liver tissues were excised and freeze-clamped with aluminum tongs precooled in liquid nitrogen. From each rat, the blood was collected to obtain serum, which was stored at −80 °C until analysis. All samples were stored immediately at −80 °C and kept frozen until further analysis.

### Determination of elongases and desaturases mRNA expression

Relative levels of specific mRNA for Elovl-2, Elovl-5, and Elovl-6 and ∆5D, ∆6D, Scd 1, and Scd 2 in rat’s liver were assessed by semi-quantitative reverse transcription polymerase chain reaction (RT-PCR). Each gene was amplified together with a housekeeping gene β-actin (Actb; internal control). Total RNA was isolated from the liver using TRI reagent (Sigma-Aldrich, Germany), and complementary DNA (cDNA) was subsequently synthesized using 1 μg total RNA, reverse-transcriptase, and oligo dT primers (RevertAid™ H Minus First Strand cDNA Synthesis Kit, Fermentas, Lithuania). cDNA was amplified with OptiTaq DNA polymerase (Eurx, Poland) following manufacturer’s instructions. Each PCR reaction was performed with 1.2 μl cDNA and 1.6 μl of specific primers for each gene (Table 1) that were designed using Primer3 v.0.4.0 (Table 1). Mastercycler gradient EP 5331 (Eppendorf, Germany) was used. The PCR products were then subjected to agarose gel electrophoresis (1.5% agarose gel), stained with ethidium bromide and analyzed by densitometry using the Quantity One 4.2.1. software (Bio Rad). Obtained data were calculated to the mean value of the 200-bp band marker first (GeneRuler™ 1 kb Plus DNA, Fermentas, Lithuania) and then normalized to β-actin (in the same sample).

**Table 1:** The primers sequences used for PCR

Gene name (symbol)	Left primer sequence	Right primer sequence	Concentration; left/right primer	Length of product
Elovl-2	TCAACAATGGCAGCTCAAAG	GGGGGATTTACTTGGGAAAA	4 μm/4 μm	249
Elovl-5	GAGGCATCCTGGTGGTGTAT	ACGTGCAGGACTGTGATCTG	4 μm/4 μm	247
Elovl-6	GCTACAACGGAGCAGAGGAC	CCATTTTCAAGCCAACCAGT	4 μm/4 μm	247
∆5D	GAAGGAACAGCAGTCCAAGC	GTCTGGACTCGTGGAAGAGC	4 μm/4 μm	185
∆6D	ATCTGCCCTACAACCACCAG	TGTGACCCACACAAACCAGT	4 μm/4 μm	248
Scd 1	CTGTTAGCCCAGCCTCACTC	GTCTGCAGGAAAACCTCTGC	2 μm/2 μm	668
Scd 2	CCAGAGCGTACCAGCTTTTC	GGCTGTCACCCAATCAGAAT	4 μm/4 μm	342
β–actin (Actb)	AGCCATGTACGTAGCCATCC	CTCTCAGCTGTGGTGGTGAA	4 μm/4 μm	228

### Lipid extraction and quantitation of hepatic FA composition

Total lipid extraction from the liver was carried out using a solution of chloroform/methanol (2:1) [26]. The synthesis of FA methyl esters (FAME) was carried out with 14% BF3 in methanol [27]. FAME were analyzed using gas chromatography (Agilent 6890 N) with FID and a DB-23 (60 m, 0.25 mm) column as described earlier [28, 29]. Fatty acid methyl esters were identified according to standards Supelco® 37 Component FAME Mix (Sigma, Supelco). The data were analyzed using ChemStation. Results were expressed in relative percentage of the sum of saturated (SAT), unsaturated (UNSAT), monounsaturated (MUFA), and polyunsaturated fatty acids (PUFA). The group of SAT includes 12:0, 14:0, 16:0, 17:0, 18:0, 21:0, and 22:0. The group of UNSAT includes 16:1 n-7, 18:1 n-9, 18:2 n-6, 18:3 n-6, 20:3 n-6, 20:4 n-6, 20:5 n-3, and 22:6 n-3. The group of MUFA includes 16:1 n-7 and 18:1 n-9. The group of PUFA includes 18:2 n-6, 18:3 n-6, 20:3 n-6, 20:4 n-6, 20:5 n-3, and 22:6 n-3.

### Desaturation, elongation, and de novo lipogenesis indexes

Particular FA ratios were used to estimate relative activities of the ∆5D (20:4n-6/20:3n-6), ∆6D (18:3n-6/18:2n-6), stearoyl-CoA desaturase 1 (SCD-1; 16:1n-7/16:0, SCD-16 and 18:1n-9/18:0, SCD-18), DNL (16:0/18:2n-6), Elovl-6 (18:0/16:0), and FA elongation (18:0 + 18:1 n-9/16:0) as described earlier [4, 30–32]. For the first time, the ratio 20:3 n-6/18:3 n-6 was used as activity of Elovl-5 (Elovl-5 index).

### Determination of rat’s serum lipid profile and glucose concentration

Serum lipid parameters including total cholesterol, HDL cholesterol, triglycerides, and glucose concentration were measured spectrophotometrically using standard kits (AQUA-MED, Poland and BioMaxima, Poland). Tests were performed according to manufacturer’s instructions. Results were presented as mmol/L.

### Statistics

Statistical calculations were performed using STATISTICA 12.0 and GraphPad Prism v. 5.02. Shapiro-Wilk test was used for normality assessing. Student’s *t* test for unpaired samples (data are presented as mean ± SD) was used to analyze mRNA expression for all elongases and desaturases, with indicators such as PUFA and MUFA, composed of 14:0, 16:0, 16:1 n-7, 17:0, 18:0, 18:1 n-9, 18:2 n-6, 18:3 n-6, 21:0, 22:0, 20:3 n-6, and 20:5 n-3, and indexes for ∆5D, Scd 1 (C16), DNL, Elovl-5, Elovl-6, elongation FAs, and glucose concentration and serum lipid profile. Mann-Whitney *U* test (data are presented as median and range) was used to analyze the indicators SAT and UNSAT, composed of 12:0, 20:4 n-6, 22:6 n-3, and indexes for ∆6D and Scd 1 (C18). Acceptable significance level was set at *p* < 0.05.

## Results

### mRNA expression of elongases and desaturases

**Fig. 1:**
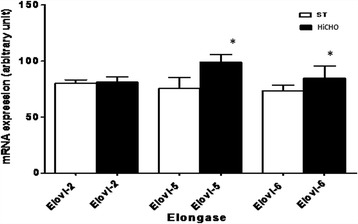
Relative levels of specific mRNA for Elovl-2, Elovl-5, and Elovl-6 in the liver of HiCHO and control groups. *HiCHO* high-carbohydrate (68%) diet-fed study group (*n* = 6), *ST rats* standard diet-fed controls (*n* = 6). Male Wistar rats weighing 151 ± 3 g were fed until achieved 250 g (21 days HiCHO vs. 25 days ST). The expression was determined by RT-PCR. Each gene was amplified together with a housekeeping gene β-actin (Actb; internal control). PCR products were separated on 1.5% agarose gel, stained with ethidium bromide, and analyzed by densitometry using the Quantity One 4.2.1. software. Obtained data were calculated to the mean value of the 200-bp band marker first and then normalized to β-actin (in the same sample). Shapiro-Wilk test was used for normality assessing. Student’s *t* test for unpaired samples; *p < 0.05

In the liver of HiCHO and ST rats, mRNA expression of Elovl-2, Elovl-5, and Elovl-6 was obtained (Fig. 1). The mean values of transcript expression of all examined elongases in liver HiCHO rats were higher than in ST. In HiCHO group, mRNA levels were increased for Elovl-2, Elovl-5, and Elovl-6 by 1, 25, and 15% as compared to ST, respectively. The difference in expression for Elovl-5 and Elovl-6 were statistically significant (*p* < 0.05).

**Fig. 2:**
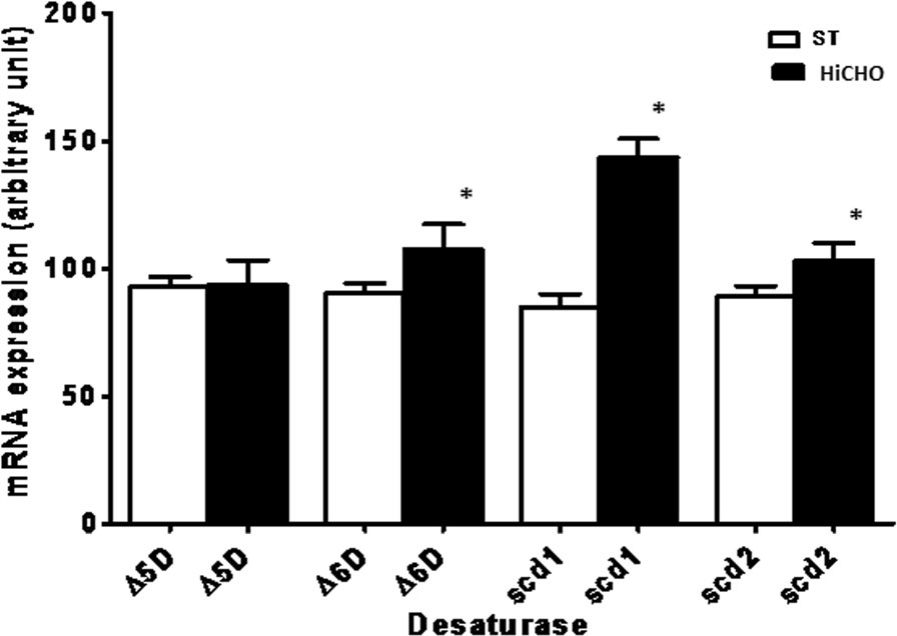
Relative levels of specific mRNA for ∆5D, ∆6D, Scd 1, and Scd 2 in the liver of the HiCHO and control groups. *HiCHO* high-carbohydrate (68%) diet-fed study group (*n* = 6); *ST rats* standard diet-fed controls (*n* = 6). Male Wistar rats weighing 151 ± 3 g were fed until achieved 250 g (21 days HiCHO vs. 25 days ST). Each gene was amplified together with a housekeeping gene β-actin (Actb; internal control). The expression was determined by RT-PCR. *p < 0.05. PCR products were separated on 1.5% agarose gel, stained with ethidium bromide, and analyzed by densitometry using the Quantity One 4.2.1. software. Obtained data were calculated to the mean value of the 200-bp band marker first and then normalized to β-actin (in the same sample). Shapiro-Wilk test was used for normality assessing. Student’s *t* test for unpaired samples; *p < 0.05

In the liver of HiCHO and ST rats, mRNA expression of ∆5D, ∆6D, Scd 1, and Scd 2 desaturases was obtained (Fig. 2). The mean values of transcript expression of all examined desaturases in liver HiCHO rats were higher than in ST. In HiCHO group, mRNA expression levels increased by 5, 19, 69, and 16% as compared to ST for desaturases ∆5D, ∆6D, Scd 1, and Scd 2, respectively. The difference in expression for ∆6D, Scd1, and Scd2 desaturases was statistically significant (*p* < 0.05).

### Hepatic fatty acid composition

**Fig. 3:**
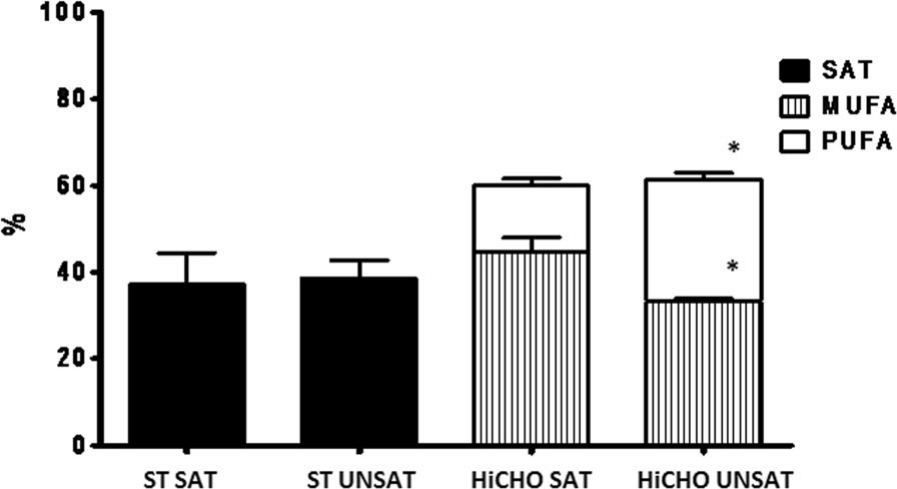
The mean values of indicators *SAT*, *UNSAT*, *PUFA*, and MUFA in the liver of the HiCHO and control groups. *HiCHO* high-carbohydrate (68%) diet-fed study group (*n* = 6); *ST rats* standard diet-fed controls (*n* = 6). Male Wistar rats weighing 151 ± 3 g were fed until achieved 250 g (21 days HiCHO vs. 25 days ST). Fatty acid composition was determined using gas chromatography. Results were expressed in relative percentage of the sum of saturated (SAT), unsaturated (UNSAT), monounsaturated (MUFA), and polyunsaturated fatty acids (PUFA). The group of SAT includes 12:0, 14:0, 16:0, 17:0, 18:0, 21:0, and 22:0. The group of UNSAT includes 16:1 n-7, 18:1 n-9, 18:2 n-6, 18:3 n-6, 20:3 n-6, 20:4 n-6, 20:5 n-3, and 22:6 n-3. The group of MUFA includes 16:1 n-7 and 18:1 n-9. The group of PUFA includes 18:2 n-6, 18:3 n-6, 20:3 n-6, 20:4 n-6, 20:5 n-3, and 22:6 n-3. Shapiro-Wilk test was used for normality assessing. Student’s *t* test for unpaired samples (data are presented as mean ± SD) was used to analyze indicators such as PUFA and MUFA; Mann-Whitney *U* test (data are presented as median and range) was used to analyze the indicators SAT and UNSAT. *p < 0.05

The mean values of SAT, UNSAT, PUFA, and MUFA in the liver of HiCHO and ST rats were calculated (Fig. 3). In the liver of HiCHO rats, the value of SAT increased by 4% and UNSAT decreased by 2.5% as compared to ST group. In liver of HiCHO group, the mean value of MUFA increased by 83% while the mean value of PUFA decreased by 26% as compared to ST. The difference in hepatic fatty acid composition for MUFA and PUFA indicators were statistically significant (*p* < 0.05).

**Fig. 4:**
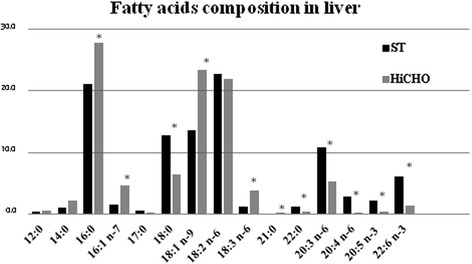
The relative percentage of individual fatty acids in the liver of the HiCHO and control groups. *HiCHO* high-carbohydrate (68%) diet-fed study group (*n* = 6); *ST rats* standard diet-fed controls (*n* = 6). Male Wistar rats weighing 151 ± 3 g were fed until achieved 250 g (21 days HiCHO vs. 25 days ST). Fatty acid composition was determined using gas chromatography. Shapiro-Wilk test was used for normality assessing. Student’s *t* test for unpaired samples (data are presented as mean ± SD) was used to analyze the composition of 14:0, 16:0, 16:1 n-7, 17:0, 18:0, 18:1 n-9, 18:2 n-6, 18:3 n-6, 21:0, 22:0, 20:3 n-6, and 20:5 n-3, and Mann-Whitney *U* test (data are presented as median and range) was used to analyze the composition of 12:0, 20:4 n-6, and 22:6 n-3; *p < 0.05

Fifteen fatty acids were determined using gas chromatography. In the liver of HiCHO and ST rats, groupwise, the relative percentage of individual fatty acids differed (Fig. 4). The relative percentage of 12:0, 14:0, 16:0, 16:1 n-7, 18:1 n-9, and 18:3 n-6 in rat liver of HiCHO group increased by 4.5, 99, 32, 207, 71, and 203%, respectively, as compared to ST. The relative percentage of 17:0, 18:0, 18:2 n-6, 22:0, 20:3 n-6, 20:4 n-6, 20:5 n-3, and 22:6 n-3 decreased by 51, 50, 3.8, 75, 50, 81%, 85.5, and 71%, respectively, as compared to ST group. Heneicosanoic acid (21:0) was detected only in the liver of HiCHO group not in ST group. The difference in the relative percentage of individual fatty acids for 16:0, 16:1 n-7, 18:0, 18:1 n-9, 18:3 n-6, 21:0, 22:0, 20:3 n-6, 20:4 n-6, 20:5 n-3, and 22:6 n-3 was statistically significant (*p* < 0.05).

### Desaturation, elongation, and DNL indexes

In the rat’s liver, the differences in the value of indexes for desaturation, elongation, and DNL were observed (Table 2). In HiCHO group, indexes for ∆5D, Elovl-5, Elovl-6, and FA elongation decreased by 80, 84, 62, and 17%, respectively, as compared to ST group. In HiCHO group, indexes for ∆6D, SCD1(16), SCD1(18), and DNL increased by 153, 137, 311, and 27% as compared to ST group. The differences in the indexes for ∆5D, ∆6D, SCD1 (16), SCD1 (18), Elovl-5, and Elovl-6 were statistically significant (*p* < 0.05).

**Table 2:** The mean values of indexes for desaturation, elongation, and DNL. Data are presented as mean ± SD or median (range)

Name	Index	ST	HiCHO
∆5D	20:4 n-6/20:3 n-6	0.25 ± 0.11	0.05 ± 0.06*
∆6D	18:3 n-6/18:2 n-6	0.07 (0.02–0.07)	0.17 (0.14–0.19)*
Scd 1 (C16)	16:1 n-7/ 16:0	0.07 ± 0.02	0.17 ± 0.02*
Scd 1 (C18)	18:1 n-9/18:0	0.92 (0.71–2.56)	3.79 (3.13–4.34)*
DNL	16:0/18:2 n-6	1.02 ± 0.36	1.29 ± 0.22
Elovl-5	20:3 n-6/18:3 n-6	9.27 ± 1.98	1.49 ± 0.43*
Elovl-6	18:0/16:0	0.60 ± 0.16	0.23 ± 0.02*
Elongation FA	18:0 + 18:1 n-9/16:0	1.3 ± 0.1	1.07 ± 0.06*

*p < 0.05

### Analysis of serum lipid profile and glucose concentration

**Fig. 5:**
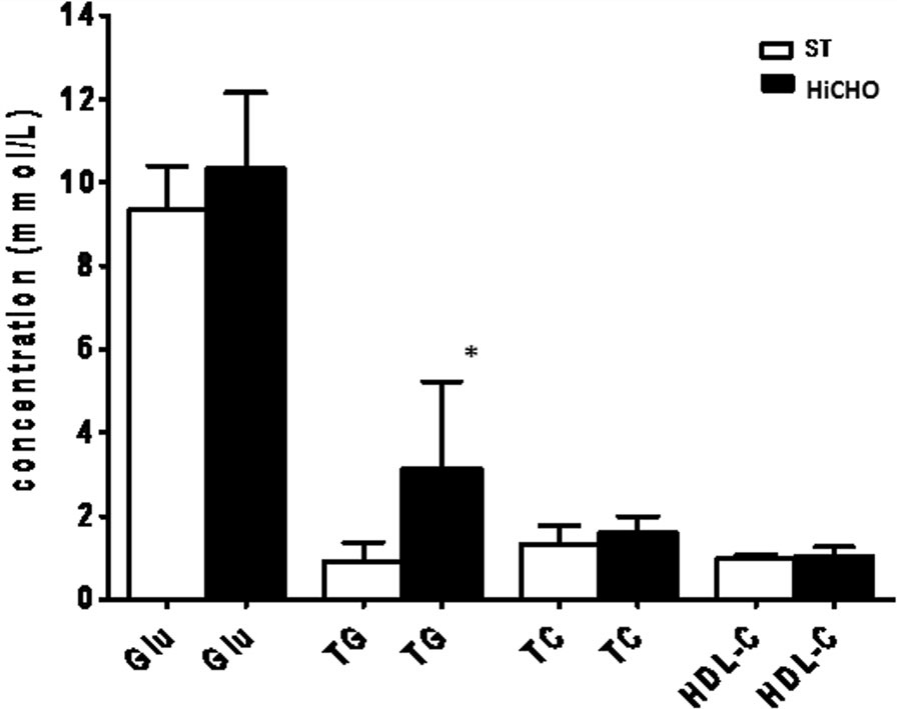
The mean concentrations of glucose, TG, TC, and HDL cholesterol in the serum of the HiCHO and control groups. *HiCHO* high-carbohydrate (68%) diet-fed study group (*n* = 6); *ST rats* standard diet-fed controls (*n* = 6). Male Wistar rats weighing 151 ± 3 g were fed until achieved 250 g (21 days HiCHO vs. 25 days ST). Shapiro-Wilk test was used for normality assessing. Student’s *t* test for unpaired samples (data are presented as mean ± SD) was used to analyze glucose concentration and serum lipid profile. *p < 0.05. Results are expressed as millimolar per liter

Parameters of serum lipid profile and glucose concentration differed in two study groups (Fig. 5). In HiCHO group, glucose, TG, TC, and HDL cholesterol increased by 11, 241, 23, and 7.4%, respectively, as compared to ST. Significantly higher concentration was only for TG (*p* < 0.05)

.

## Discussion

### De novo FA synthesis and MUFA

Diets enriched in carbohydrates are well established to alter FA metabolism and increase TG accumulation [4]. In our study, we observed the active DNL and the influence of the HiCHO diet on increased expression of all studied enzymes resulting in increased production of MUFAs. The most important changes concern metabolism of 16:0 and 18:0 as a dominant products of DNL [30]. Both FAs are subjects to elongation or desaturation processes [33]. On the basis of our observation, the desaturation is a main process of 16:0 and 18:0 metabolism in liver of rat fed HiCHO diet because of a higher expression of mRNA for Scd1 (Fig. 2), a higher content of 16:1 n-7 and 18:1 n −9 (Fig. 4), and a higher activity of desaturases as indexes for Scd1 (C16) and Scd1 (C18) (Tab. 2) as compared to control group. The index for Scd1 (C16) is proposed as an indirect biomarker of DNL [30]. Scd 1 is the main isoform in MUFAs synthesis with 16:0 and 18:0 as main substrates [34]. As Scd1 plays a crucial role in this synthesis, the role of Scd2 remains unclear. Our findings indicate that 16:0 and 18:0 metabolism is directed to MUFAs synthesis as 16:1 n-7 and 18:1 n-9, respectively, that are main components of TG and PL. Especially, 18:1 n-9 is the preferred substrate for TG storage of excess FAs [4]. In our study, it was observed that in HiCHO group, liver tissues contain statistically more MUFAs (with prevalence 16:1 n-7 and 18:1 n-9 fatty acids; Figs. 3 and 4) and less PUFAs than control. The study by Wang et al. [1] indicates that 16:0, 18:0, 18:1 n-9, and 18:2 n-6 acids represent the major fatty acids and 16:1 n-7 is an end product of DNL and Scd. The results from our study (Fig. 4) are consistent with the results obtained by Wang et al. [1]. The last but not the least, in our study, serum level of TG was higher in HiCHO than in control (Fig. 5) that may indicate that newly synthesized FAs as TG incorporated into lipoproteins were released to plasma.

The glucose is converted into FAs in DNL process by the induction of genes transcription that increase glucose metabolism and lipogenesis in the liver [4]. According to substrate specific, Elovl-6, Scd1, and Scd2 desaturases might be responsible for these changes [2, 8]. Genes of Elovl-6, Scd1, and Scd2 desaturases contain the response element for transcriptional factors such as SREBP, LXR, and ChREBP, in their promoter [1]. Wang et al. [1] indicate that in primary hepatocytes glucose regulates both Elovl-6 and Scd expression by mechanisms that control the nuclear abundance of ChREBP and MLX. It is known that during carbohydrates excess, glucose/insulin stimulates hepatic lipogenesis via LXR and SREBP-1 [18, 35, 36]. Glucose as natural ligand for LXR may induce FA and TG synthesis as it was seen in our study, where rat fed with HiCHO diet had higher serum TG level than control group. ChREBP, a central transcriptional regulator of hepatic DNL, is also regulated by glucose itself, increasing expression of mRNA for elongase 6 and Scd desaturases [1, 2].

### PUFA synthesis

Metabolism of PUFAs depends on the content of 18:2 n-6 and 18:3 n-3 as essential FAs derived from a diet. These FAs are substrates for elongases and desaturases. In our study, we observed that liver tissues of HiCHO group contain statistically less PUFAs than control group (Fig. 3). In the current study, it was observed that the diet enriched in glucose increased expression of Elovl-5, ∆5D, and ∆6D mRNA (Figs. 1 and 2) but relative activities of these enzymes were different (Tab. 2). The result of these processes is reduction of PUFA synthesis. Because of the low activity of Elovl-5 and ∆5D, we observed the lower content of 20:3 n-6, 20:4 n-6, and 22:6 n-3 PUFAs in HiCHO group than in control group. The control mechanism of decreasing activity of these two enzymes remains unresolved. Moon et al. [10] reported that ablation of Elovl5 increases hepatic lipid content, at least in part by inducing SREBP-1, a key transcription factor controlling de novo lipogenesis. Tissues of Elovl5(−/−) mice accumulated the C18 substrates of ELOVL5 and the levels of C20:4 n-6 and C22:6 n-3 were decreased [10]. The same changes in the content of FAs were observed in our study but mainly as a result of lower relative activity of Elovl-5 than the lower level of expression. It cannot be excluded that high expression of SREBP-1c decrease activity of Elovl-5. It may explain the results achieved in the current study because of glucose as a strong inducer of SREBP-1. Recently, Wang et al. [37] reported the metabolic effects of adenoviral-mediated overexpression of Elovl-5. The relative levels of 20:4 n-6 and 22:6 n-3 in mouse livers that overexpressed Elovl-5 were decreased despite an increase in 20:3 n-6. Thus, it is possible that the ∆5 desaturase becomes rate-limiting in case of Elovl-5 overexpression [37]. ∆5D a rate-limiting enzyme, as it was suggested by Wang, can explain only low levels for 20:4 n-6 and 22:6 n-3. More research is needed to clarify the cause of Elovl-5 and ∆5D low activity.

In the current study, only two members of n-3 PUFA family were determined (20:5 n-3 and 22:6 n-3; Fig. 4). It is difficult to explain changes in content of these FAs in the liver because of the lack of 18:3 n-3 precursor. We may conclude that the hypothesis stated by Wang [37] (∆5 desaturase is a rate-limiting enzyme) may explain lower hepatic levels of 20:5 n-3 and 22:6 n-3 in HiCHO group compares to ST.

The expressions of Elovl-5, ∆5D, and ∆6D involved in PUFA metabolism are under control of transcription factors such as PPAR, LXR, and SREBP-1c [1, 19, 35, 38]. Genes of Elovl-5, ∆5D, and ∆6D contain the response element for these transcriptional factors in their promoter. Only elongase 2 is not regulated in this way. In our study, expression of elongase 2 did not change in liver of rats fed high carbohydrate diet in comparison to control group. Our results complements data obtained by Wang et al. [3], who investigated the influence of different diets (high carbohydrate diet supplemented with olive oil, fish oil, or olive oil plus WY14,643) on mRNA expression for elongase 2. None of them changed the mRNA expression of elongase 2 [3]. Hepatic Elovl-5 is regulated by LXRα-SREBP-1c [19]. Mitro et al. [18] showed that glucose binds and stimulates the transcriptional activity of LXR. These findings may suggest the regulation of Elovl-5 expression by these factors in liver of rats fed high carbohydrate diet. The problem of low activity of Elovl-5 remains unresolved. Wang et al. [1] suggest that the induction of ∆5D and ∆6D by insulin and T1317 (the LXR agonist) is attributable to increased SREBP-1 nuclear abundance. These findings, altogether, may explain higher expression of ∆5D and ∆6D in livers of rats of HiCHO group as compared to control group. PPAR-α expressed primarily in the liver is essential for metabolic adaptation to starvation by inducing genes for β-oxidation and ketogenesis and by downregulating energy expenditure [39]. It can confirm that this transcriptional factor through a high carbohydrate diet given ad libitum plays a minor role in regulation of lipogenic gene transcription. To sum up, increased hepatic glucose flux activates SREBP and LXR, which act to couple increased carbohydrate intake with induction of lipogenic genes as ∆5D and ∆6D.

## Conclusions

In this study, we have established that hepatic fatty acid elongases (Elovl-5 and Elovl-6) and desaturases (∆5D, ∆6D, Scd-1, and Scd-2) are regulated by high carbohydrate diet. Additionally, we have indicated that changes in activity do not always correspond to changes of Elovl and desaturases mRNA levels. We have demonstrated that elevated intake of glucose modified FAs composition in liver with prevalence of MUFA as well as increased serum level of TG, of which MUFA are main components. The changes might be connected with unfavorable lipogenesis in liver. These studies support the notion that the regulation of both Elovl and desaturase expression may play an important role in managing hepatic lipid composition in response to changes in dietary status.

## Abbreviations

∆5D: ∆5 desaturase
∆6D: ∆6 desaturase
∆9D or SCD: ∆9 desaturase
ChREBP: Carbohydrate-responsive element-binding protein
DNL: De novo lipogenesis
Elovl: Elongases
ER: Endoplasmic reticulum
FAME: Fatty acid methyl esters
FAs: Fatty acids
KAR: 3-ketoacyl-CoA reductase
LXRα: Liver X receptor α
MUFA: Monounsaturated fatty acids
PL: Phospholipids
PUFA: Polyunsaturated fatty acids
RT-PCR: Reverse transcription polymerase chain reaction
SAT or SFA: Saturated fatty acids
SCD1: Stearoyl-CoA desaturase isoform 1
SREBP-1c: Sterol regulatory element-binding proteins 1c
TC: Total cholesterol
TER: 2,3-transenoyl-CoA reductase
TG: Triglycerides
UNSAT: Unsaturated fatty acids
